# Proliferative and anti-proliferative effects of dietary levels of phytoestrogens in rat pituitary GH_3_/B_6_/F_10 _cells - the involvement of rapidly activated kinases and caspases

**DOI:** 10.1186/1471-2407-9-334

**Published:** 2009-09-18

**Authors:** Yow-Jiun Jeng, Cheryl S Watson

**Affiliations:** 1 Department of Biochemistry and Molecular Biology, University of Texas Medical Branch, Galveston, Texas, USA

## Abstract

**Background:**

Phytoestogens are a group of lipophillic plant compounds that can have estrogenic effects in animals; both tumorigenic and anti-tumorigenic effects have been reported. Prolactin-secreting adenomas are the most prevalent form of pituitary tumors in humans and have been linked to estrogen exposures. We examined the proliferative effects of phytoestrogens on a rat pituitary tumor cell line, GH_3_/B_6_/F_10_, originally subcloned from GH_3 _cells based on its ability to express high levels of the membrane estrogen receptor-α.

**Methods:**

We measured the proliferative effects of these phytoestrogens using crystal violet staining, the activation of several mitogen-activated protein kinases (MAPKs) and their downstream targets via a quantitative plate immunoassay, and caspase enzymatic activities.

**Results:**

Four phytoestrogens (coumestrol, daidzein, genistein, and *trans*-resveratrol) were studied over wide concentration ranges. Except *trans*-resveratrol, all phytoestrogens increased GH_3_/B_6_/F_10 _cell proliferation at some concentration relevant to dietary levels. All four phytoestrogens attenuated the proliferative effects of estradiol when administered simultaneously. All phytoestrogens elicited MAPK and downstream target activations, but with time course patterns that often differed from that of estradiol and each other. Using selective antagonists, we determined that MAPKs play a role in the ability of these phytoestrogens to elicit these responses. In addition, except for *trans*-resveratrol, a serum removal-induced extrinsic apoptotic pathway was blocked by these phytoestrogens.

**Conclusion:**

Phytoestrogens can block physiological estrogen-induced tumor cell growth *in vitro *and can also stimulate growth at high dietary concentrations in the absence of endogenous estrogens; these actions are correlated with slightly different signaling response patterns. Consumption of these compounds should be considered in strategies to control endocrine tumor cell growth, such as in the pituitary.

## Background

**Figure 1:**
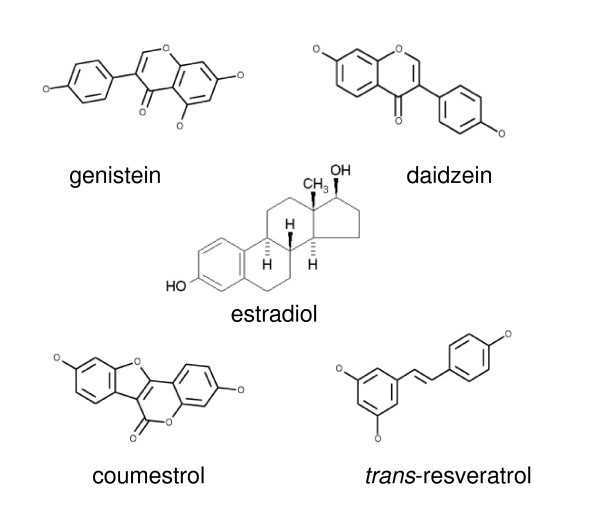
**The structure of estradiol compared to phytoestrogens**. Chemical structures of the phytoestrogenic compounds used in this study are presented, compared to the physiological estrogen E_2_.

Phytoestrogens are defined functionally as plant-derived compounds that promote estrogenic actions in mammals, and are somewhat structurally similar to the potent mammalian estrogen, 17β-estradiol (E_2_) (see Figure 1). The diverse biological activities of phytoestrogens may be due in part to their ability to act as either estrogen agonists or antagonists, depending on the dose and the specific tissue in which they are active. These abilities have caused a lot of attention to be focused on these compounds as potential safe, effective, and inexpensive estrogen replacement therapeutics. Estrogens can influence the growth and functioning of both female and male reproductive tissues, maintain the skeletal and central nervous systems, and provide protective effects in the cardiovascular system [1]. However, prolonged estrogen exposures have also been linked to the development of cancer in tissues such as breast, colon, and pituitary [2-4]. So it is important to determine which of these estrogenic attributes are shared by phytoestrogens.

Phytoestrogens have been shown to bind to two types of estrogen receptors (ERs, α and β) [5,6] and perhaps also to act via the alternative ER, GPR30 [7], Although this class of estrogens can elicit effects via the genomic pathway, those nuclear actions have in the past been described as weak for most phytoestrogens. We instead hypothesized that some actions of phytoestrogens relevant to control of cell numbers could be via plasma membrane forms of these receptors, which use rapid enzymatic cascades and ion fluxes to elicit relatively rapid functional responses. These actions have, however, remained largely untested. Here we asked these questions in a cell line selected for its high level of membrane ERα expression, and its ability to display a variety of rapid estrogenic responses [8-10]. However, it is probable that both types of mechanistic pathways (nuclear and membrane) are involved in a function as complex as cell proliferation.

For these experiments we examined three different types of phytoestrogens and their effects on pituitary tumor cell growth. The first group is coumestans, represented in our studies by coumesterol. Coumesterol was first reported to be estrogenic in 1957 [11] when it was associated with disrupting reproduction in livestock. It is found in dietary sources such as legumes, clover, and sprouts of soybeans and alfalfa. The reported average serum concentration resulting from ingesting these foods in humans is approximately 0.01 μM [12]. The second group is isoflavones, represented by daidzein and genistein in our experiments. The major source of isoflavonoids in the diet is soy-based foods. In Asia, the intake of soy can be as high as 30-50 g a day and plasma concentrations of genistein from 0.1 to 10 μM have been measured [12,13]. Western diets usually contain about ten-fold lower concentrations [14]. We also studied *trans*-resveratrol, a stilbene, which is found in high quantities in foods such as red grapes (wine) and peanuts. There are two isomers of resveratrol, *cis *and *trans*, but only the *trans *form has been reported to be estrogenic [15]. *Trans*-resveratrol has high bioavailability and relatively high physiological levels can be obtained through drinking red wine. The peak serum concentration of *trans*-resveratrol and its metabolites was estimated to be close to 2 μM in humans [16]. All of these phytoestrogens in our studies have been shown to have estrogenic and anti-estrogenic effects, depending on the types and developmental status of tissues.

Prolactinomas develop from prolactin-secreting lactotropes in the anterior pituitary gland and are the most frequently occurring neoplasm of the human pituitary. Hyperprolactinemia can cause reproductive dysfunctions such as amenorrhea, galactorrhea and infertility in women, and impotence and reduced reproductive hormones levels in men. It is also associated with some behavioral disturbances [17,18]. Estrogen exposures are linked to prolactinomas in both animals and humans. Women using oral contraceptives due to menstrual irregularities show a 7-8 fold higher incidence of prolactinomas, and male transsexuals undergoing sex change therapies also have a higher incidence of these tumors [4]. Subcutaneous implants of diethylstilbestrol cause pituitary hyperplasia and neoplasms in Fischer F344 rats [19,20]. Pituitaries also normally respond to estrogens by undergoing cell proliferation in response to pregnancy level estrogens; under these conditions the number of prolactin-secreting cells and serum prolactin content increases. Although the exact mechanism by which estrogens increase mitogenesis is not completely understood, this increased proliferation response can sometimes result in prolactinomas. Increased production and secretion of prolactin, and induction and activation of cell cycle regulatory proteins leading to a more rapid rate of cell division may eventually lead to uncorrected genetic errors, resulting in the activation of oncogenes or inactivation of suppressor genes, genomic instability, and transformation [21].

Mitogen-activated protein kinases (MAPKs) control many aspects of mammalian cellular physiology, including cell growth, differentiation and apoptosis, and they respond to a variety of mitogens and growth factors. They can also phosphorylate downstream targets such as Elk-1 and ATF-2 transcription factors [22,23]. Activating these downstream targets of MAPKs can influence chromatin remodeling and gene expression affecting cell proliferation [24,25]. There are three major MAPK types including extracellular signal-regulated kinases (ERKs), c-JUN N-terminal kinases (JNKs) and p38 kinases. Recently, we found that estrogens (both physiological and environmental) can rapidly activate ERKs via membrane ERα (mERα) and non-genomic pathways in GH_3_/B_6_/F_10 _cells [26]. Therefore, we became interested in the possibility that phytoestrogens may also activate MAPKs, and play a role in how estrogens elicit or inhibit cell proliferation.

Due to the possible undesirable side effects of estrogenic stimulation (such as increases in tumor risks), many women have turned to phytoestrogens as an alternative for hormone replacement therapy. This is based on the idea that in Asian cultures where these dietary estrogens are present in much higher exposure levels than in Western diets, that the diseases attributable to estrogen loss (hot flashes and bone, brain, and cardiovascular tissue decline) are significantly less evident, and the diseases of estrogen overexposure (cancer of reproductive tissues) are also significantly lower. Therefore, it has been reasoned that these plant estrogens may be safe and effective replacements that do not increase cancer risks. There is a rapidly growing body of literature on the functional consequences of phytoestrogen use, and the biochemical characteristics of the compounds, their metabolites, and their cellular response systems, especially for soy products. But, little investigation has thus far addressed the question of whether phytoestrogens can cause pituitary tumor cell growth, and if so, via what nongenomic mechanisms this might occur.

## Methods

We purchased phenol red-free Dulbecco modified Eagle medium (DMEM, high glucose) from Mediatech (Herndon, VA); horse serum from Gibco BRL (Grand Island, NY); defined supplemented calf sera and fetal bovine sera from Hyclone (Logan, UT). Paraformaldehyde and glutaraldehyde were purchased from Fisher Scientific (Pittsburgh, PA). Antibodies used in the measurement of levels of phosphorylaed MAPKs and their downstream targets were purchased from Cell Signaling Technology (Danvers, MA). Kinase phospho-antibodies recognize more than one member of each family of kinase. The MAPK inhibitors, and the substrates and blockers for caspase assays were purchased from Biomol (Plymouth Meeting, PA). All other materials were purchased from Sigma (St. Louis, MO).

GH_3_/B_6_/F_10 _cells are routinely cultured in DMEM containing 12.5% horse serum, 2.5% defined supplemented calf serum, and 1.5% fetal calf serum. They are then switched to various hormone-free media prior to our experiments, as described below. Cells were used between passages 10 to 20.

### Proliferation assays

Subconfluent cells growing in serum-containing medium were seeded into 96-well plates coated with poly-D-lysine (5000 cells/well), allowed to attach overnight, and treated with E_2 _or various phytoestrogens in DMEM medium containing 1% 4× charcoal-stripped FBS for 3 days. Cell numbers were assessed by the crystal violet assay to compare the proliferative effects of phytoestrogens at different concentrations. On the day of cell number analysis, cells from which medium had been removed were fixed for 20 min in 2% paraformaldehyde and 0.1% glutaraldehyde in PBS. They were then stained for 30 min with a 0.1% solution of crystal violet, and destained in deionized water. The dye was released with 10% acetic acid at RT for 30 min, and the A_590 _signal of the extract was then read in a model 1420 Wallac microplate reader (Perkin Elmer, Waltham, MA). We used 8 samples for each treatment group, and experiments were repeated 3 times using different passages of cells.

### MAPKs, Elk-1, and ATF-2 assays

We originally developed this assay to assess activated ERK 1 and 2 levels in fixed GH_3_/B_6_/F_10 _cells [10] and subsequently adapted it to equivalent assays for p38s and JNKs, and now in this study for the phosphorylated transcription factors Elk-1 and ATF-2. Briefly, cells were plated at a density of 10,000 cells/poly-D-lysine-coated well in 96-well plates. The following day growth media were replaced with DMEM containing 1% charcoal-stripped (4×) serum (to deprive cells of steroids) for 48 hr. Cells were then washed with DMEM once before the E_2 _or the phytoestrogens (or 0.0001% ethanol vehicle) were added for 2.5 to 60 min. We routinely used cholesterol as a negative control and phorbol 12-myristate 13-acetate as a positive control. The cells were then fixed with 2% paraformaldehyde/0.2% picric acid at 4°C for 48 hr. After fixation, the cells were permeabilized with PBS containing 2% BSA and 0.1% Triton X-100 for 1 hr at RT, washed 3× with PBS, and primary antibody (Ab) against phosphorylated ERKs (p-Thr202/Tyr204; 1:400 in PBS/1% BSA), phosphorylated JNK (p-Thr183/Tyr185; 1:500 in PBS/1% BSA), phosphorylated p38 (p-Thr180/Tyr182; 1:500 in PBS/1% BSA), phosphorylated Elk-1 (p-Ser383; 1:750 in PBS/1% BSA), or phosphorylated ATF-2 (p-Thr69/71, 1;500 in PBS/1%BSA) was added. After overnight incubation at 4°C, the cells were washed 3× with PBS, and the biotin-conjugated secondary Ab (Vector Labs, Burlingame CA, 1:300) in PBS/1% BSA was added for 1 hr at RT. The cells were again washed with PBS, incubated with Vectastain ABC-AP solution (Vector Labs) for 1 hr at RT, and again washed 3× with PBS, followed by addition of Vectastain alkaline phosphatase substrate plus levamasole (an endogenous phosphatase inhibitor). Plates were incubated in the dark for 30 min at 37°C, and the signal for the phosphatase product *para*-nitrophenol was read at A_405 _in a model 1420 Wallace microplate reader. The number of cells in each well was determined by the crystal violet assay (as described above) and used to normalize phosphorylated enzyme values to cell numbers in individual wells. The experiments were repeated at least 3 times using different passages of cells on different days.

### Caspase activity assays

Subconfluent cells growing in serum-containing medium were seeded into 96-well plates coated with poly-D-lysine (5000 cells/well), allowed to attach overnight, and treated with E_2 _or various phytoestrogens in DMEM medium containing 1% 4× charcoal-stripped FBS for 1 day. Cells were spun down, washed with ice-cold PBS, and 100 μl of lysis buffer (10 mM Hepes, pH 7.4, 2 mM EDTA 0.1% CHAPs), to which 5 mM DTT and proteinase inhibitor (from Sigma) were added. To perform the caspase activity assay, 50 μl of the cell lysates were then mixed with 50 μl of caspase assay buffer (50 mM Hepes, pH 7.4, 100 mM NaCl, 0.1% CHAPs, 1 mM EDTA, 10% glycerol, 10 mM DTT) containing 50 μM of substrate Ac-IETD-AFC (specific for caspase-8) or Ac-LEHD-AFC (specific for caspase-9), and incubated at 37°C in the dark. The enzymes catalyzed release of a fluorescent product (AFC) which was monitored at times 0 and 1 hr using a spectrofluorometer (GeminiXS, Molecular Devices) at an excitation wavelength of 400 nm and an emission wavelength of 505 nm. The cell number in each sample are measured by the crystal violet assay and showed no significant changes at different time points and treatments. The caspase activity is expressed as change in fluorescence intensity during an hr for 2500 cells.

### Statistics

Data from cell number measurements, and MAPK and transcription factor assays were analyzed by one-way analysis of variance (ANOVA) followed by multiple comparisons versus control group (Holm-Sidak method). The Sigma Stat 3 program (Systat Software, Inc.) was used for all statistical analysis, and significance was accepted at p < 0.05.

## Results

### Phytoestrogens change the proliferation of GH_3_/B_6_/F_10 _cells

**Figure 2:**
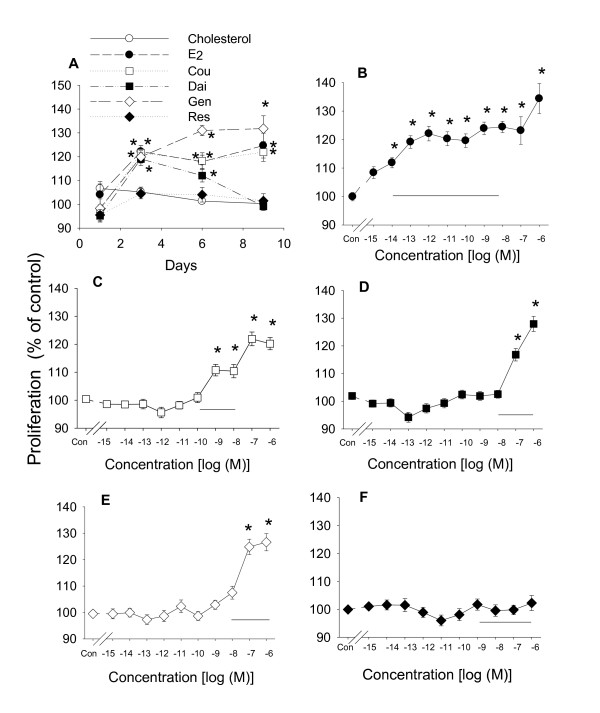
**Proliferative effects of E_2 _and phytoestrogens on GH_3_/B_6_/F_10 _cells**. (A) Cells treated with physiological or dietary levels of estrogens: 10^-9 ^M E_2_, 10^-8 ^M coumesterol (Cou), 10^-7 ^M cholesterol (negative control), daidzein (Dai), genistein (Gen), or *trans*-resveratrol (Res) over a 9 day time course. Cell proliferation was measured by the crystal violet assay. (B-F) Dose-response profiles of the proliferative effects of different concentrations of estrogens after 3 days of treatment. (B) E_2_, (C) coumesterol, (D) daidzein, (E) genistein, and (F) *trans*-resveratrol. The solid horizontal line spans the reported concentrations of each estrogen in human serum or urine. * = p < 0.05 compared to vehicle-treated cells.

We first determined the proliferative effects of different phytoestrogens in GH_3_/B_6_/F_10 _cells over a 9 day time course, compared to E_2_, to choose a day when the differential effects of the compounds were clearly manifested. We chose the concentrations for growth analyses based on the published dietary concentrations of these compounds, and compared the effects to those caused by cholesterol, the structurally similar precursor for all steroid hormones (which did not induce a growth response compared to the ethanol vehicle control). A horizontal bar on each graph (Figure 2, B-F) shows an estimate of the dietary concentration ranges for these compounds that have been measured in serum. Most compounds demonstrated statistically maximal proliferative effects by day 3 (Figure 2A) in these extensively hormone-stripped serum conditions. Comparable proliferative activities were caused by 10^-9 ^M E_2_, 10^-8 ^M coumesterol, 10^-7 ^M daidzein and 10^-7 ^M genistein on day 3. Daidzein caused a less robust response on subsequent days. *Trans*-resveratrol and cholesterol at a concentration of 10^-7 ^M showed no proliferative effects on any of the days tested. We then examined the potency with which each compound caused proliferation on day 3, in comparison to E_2_. E_2 _significantly increased cell number at a wide range of concentrations from 10^-14 ^to 10^-6 ^M (Figure 2B). Coumestrol caused proliferation from 10^-9 ^to 10^-6 ^M concentrations (Figure 2C). Weaker effects were observed for daidzein and genistein, both of which increased cell number only with high concentrations of 10^-7 ^and 10^-6 ^M (Figure 2D and 2E), which are still however at levels obtainable by ingestion of these compounds in a typical Asian human diet. *Trans*-resveratrol did not cause cell proliferation at any concentration examined (Figure 2F).

### Phytoestrogens attenuate the proliferative effects of E_2_

**Figure 3:**
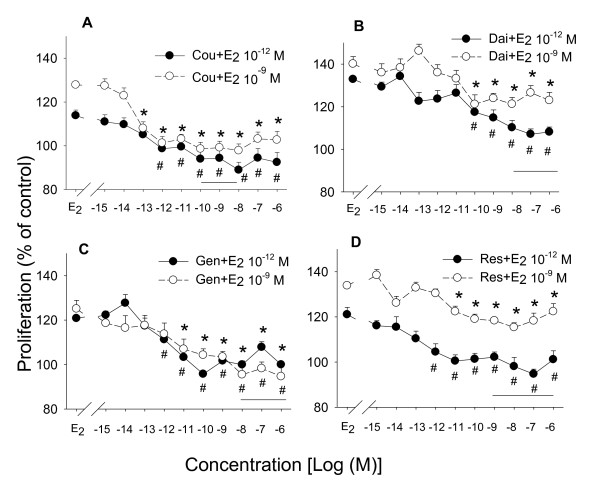
**Proliferative effects of E_2 _in combination with different concentrations of phytoestrogens**. GH_3_/B_6_/F_10 _cells were treated with different concentrations of phytoestrogens in the presence of either 10^-9 ^M or 10^-12 ^M E_2_. The cell numbers were measured after 3 days of treatment. (A) coumesterol (B) daidzein (C) genistein (D) *trans*-resveratrol. The solid horizontal line spans the reported concentrations of each estrogen in human serum or urine. * = p < 0.05 compared to 10^-9 ^M E_2_. # = p < 0.05 compared to 10^-12 ^M E_2_.

To examine the anti-proliferative effects of phytoestrogens, we co-treated the GH_3_/B_6_/F_10 _cells with either of two concentrations representing low and high endogenous levels (10^-12 ^M or 10^-9 ^M) of E_2_, plus different concentrations in log increments of coumesterol, daidzein, genistein, or *trans*-resveratrol (Figure 3). Coumesterol showed the most anti-proliferative effects, all concentrations higher than 10^-12 ^M blocking the effects of 10^-12 ^or 10^-9 ^M of E_2 _(Figure 3A). Daidzein at concentrations ≥10^-10 ^M attenuated proliferative effects of either 10^-12 ^or 10^-9 ^M E_2_, but the proliferative effects of 10^-9 ^M E_2 _were not completely blocked (Figure 3B). Genistein at 10^-11 ^to 10^-6 ^M concentrations attenuated the proliferative effects of both 10^-12 ^or 10^-9 ^M of E_2 _(Figure 3C). *Trans*-resveratrol concentrations higher than 10^-12 ^M blocked the proliferative effects of 10^-12 ^M E_2 _completely, but similar to daidzein, *trans*-resveratrol only decreased the cell number by about 15% when combined with 10^-9 ^M E_2 _(Figure 3D). Interestingly, the anti-proliferative effects of phytoestrogens require lower concentrations than the proliferative effects. Compared to the expected dietary levels seen in Asian diets (see horizontal bars), these proliferation-inhibiting effects might reasonably be expected to be seen with concentrations obtainable by eating foods containing them.

### Phosphorylation of MAPKs by E_2 _and different phytoestrogens

**Figure 4:**
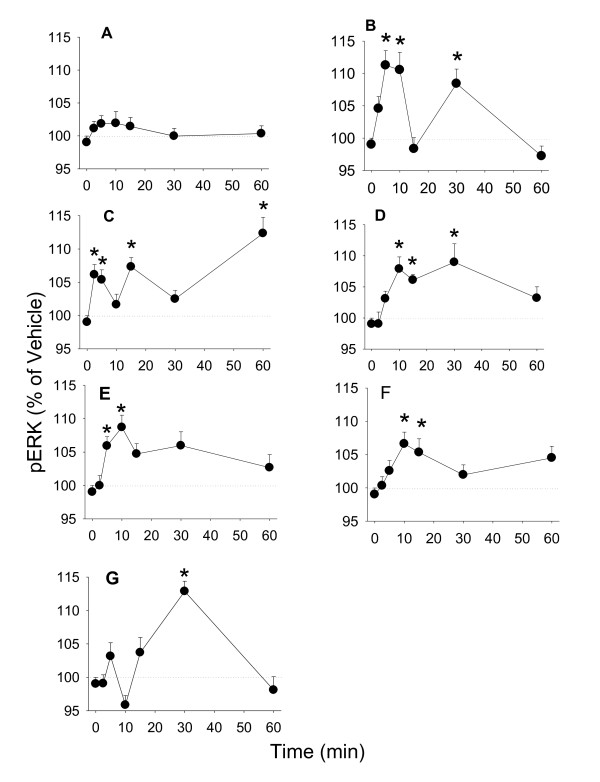
**The time course of estrogenic phospho-activation of ERK**. Time-dependent activation of ERK measured by a quantitative plate immunoassay in cells treated with (A) 10^-7 ^M cholesterol (negative control), (B) 20 nM TPA (positive control), (C) 10^-9 ^M E_2_, (D) 10^-8 ^M coumesterol, (E) 10^-7 ^M daidzein, (F) 10^-7 ^M genistein, (G) 10^-7 ^M *trans*-resveratrol. * = p < 0.05 compared to vehicle only treated cells.

The most frequently reported and clearly defined pathway for the rapid action of estrogens is the ERK pathway [10,27-29]. Cholesterol did not induce a significant ERK activation across this time course at a 10^-7 ^M concentration (Figure 4A); TPA caused the expected robust ERK activation at early (5-10 min) and late (30 min) time points (Figure 4B). E_2 _at a concentration of 10^-9 ^M caused a rapid activation of ERK (significant at 2.5 min). Then the response decreased, followed by an additional activation at 15 and again at 60 min (Figure 4C), similar to the oscillating activation that we observed previously [10,26,30]. Coumesterol at a concentration of 10^-8 ^M caused a more sustained activation of ERK, maintaining significance at 10 to 30 min (Figure 4D), also similar to previous observations [26]. Daidzein at a 10^-7 ^M concentration increased pERK at 5-10 min (Figure 4E), and genistein at this concentration at 10-15 min (Figure 4F). *Trans*-resveratrol at a 10^-7 ^M concentration caused a significant increase of pERK at 30 min only (Figure 4G).

### Phosphorylation of other MAPKs

**Figure 5:**
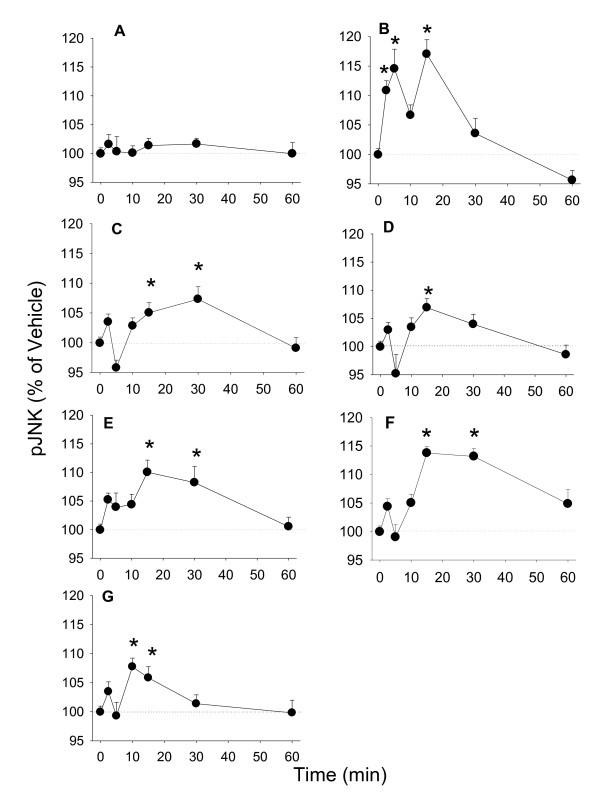
**The time course of estrogenic phospho-activation of JNK**. Time-dependent activation of JNK measured by a quantitative plate assay in cells treated with (A) 10^-7 ^M cholesterol (negative control), (B) 20 nM TPA (positive control), (C) 10^-9 ^M E_2_, (D) 10^-8 ^M coumesterol, (E) 10^-7 ^M daidzein, (F) 10^-7 ^M genistein, (G) 10^-7 ^M *trans*-resveratrol. * = p < 0.05 compared to vehicle only treated cells.

**Figure 6:**
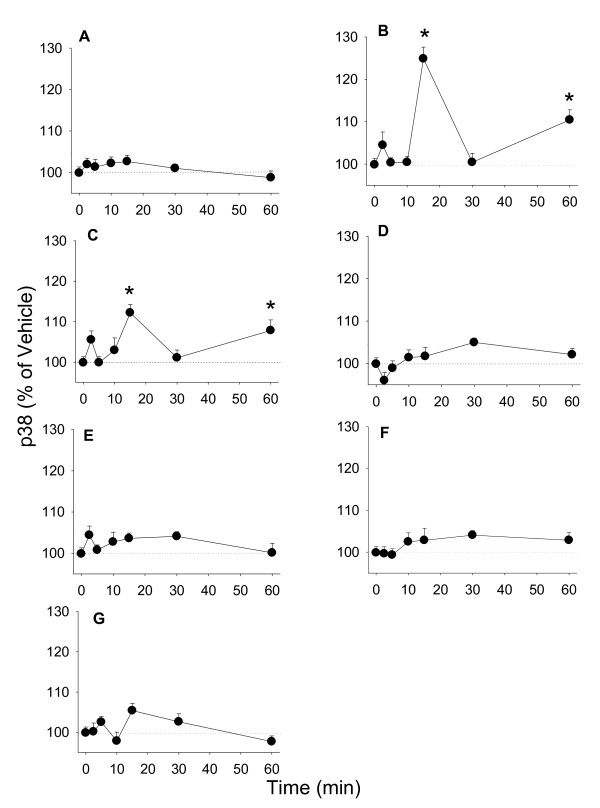
**The time course of estrogenic phospho-activation of p38**. Time-dependent activation of p38 measured by a quantitative plate assay in cells treated with (A) 10^-7 ^M cholesterol (negative control), (B) 20 nM TPA (positive control), (C) 10^-9 ^M E_2_, (D) 10^-8 ^M coumesterol, (E) 10^-7 ^M daidzein, (F) 10^-7 ^M genistein, (G) 10^-7 ^M *trans*-resveratrol. * = p < 0.05 compared to vehicle only treated cells.

Other MAPKs such as JNK and p38 can also regulate cell proliferation, though their exact roles are debated as to whether they are proliferative or apoptotic [31]. We measured time-dependent phosphorylation of JNKs and p38s in cells treated with E_2 _or phytoestrogens. Between 10 and 30 min, all estrogens increased levels of phosphorylated JNK significantly (Figure 5) with slightly different peak times. As for p38, only 10^-9 ^M E_2 _increased phosphorylation of p38 at 15 and 60 min significantly; none of the phytoestrogens changed phosphorylated p38 levels significantly (Figure 6). TPA activated both JNKs and p38s as expected.

### Signaling pathways involved in the proliferative effects of E_2 _and different phytoestrogens

**Figure 7:**
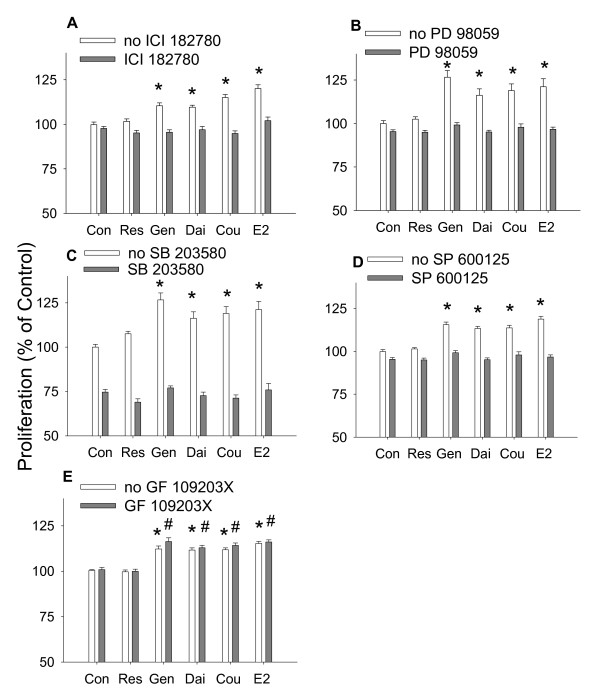
**The effects of different antagonists on the proliferative effect of phytoestrogens**. The proliferative effects of 10^-9 ^M E_2_, 10^-8 ^M coumesterol, 10^-7 ^M daidzein, 10^-7 ^M genistein and 10^-7 ^M *trans*-resveratrol in the presence signaling molecule antagonists for: (A) ERα/β (ICI 182780, 1 nM) (B) ERKs (PD 98059, 10 μM) (C) p38 (SB203580, 1 μM) (D) JNK (SP 600125, 5 μM) (E) PKC (GF 109203×, 10 μM, negative control). * = p < 0.05 compared to control cells treated with vehicle only. # = p < 0.05 compared to cells treated with antagonists only.

To investigate whether ER α/β were involved in the proliferative effects of phytoestrogens, we co-treated the cells for 3 days with 1 nM ICI 182,780 (which depletes cellular ER levels, [32]) and each estrogen separately: 10^-9 ^M E_2_, 10^-8 ^M coumesterol, and 10^-7 ^M daidzein, genistein, and *trans*-resveratrol. ICI 182,780 successfully blocked the proliferative effects of all phytoestrogens (Figure 7A) except *trans*-resveratrol which showed no proliferative effects. ERK antagonist (PD 98059, 10 μM), p38 antagonist (SB203580, 1 μM), and JNK antagonist (SP 600125, 5 μM) all attenuated the proliferative effect of all phytoestrogens examined (Figure 7B-D). The PKC antagonist (GF 109203X, 10 μM) did not change the proliferative effects of any of these phytoestrogens (Figure 7E) suggesting that these effects are selective for MAPK signaling pathways.

### Caspase activity evoked by E_2 _and different phytoestrogens

**Figure 8:**
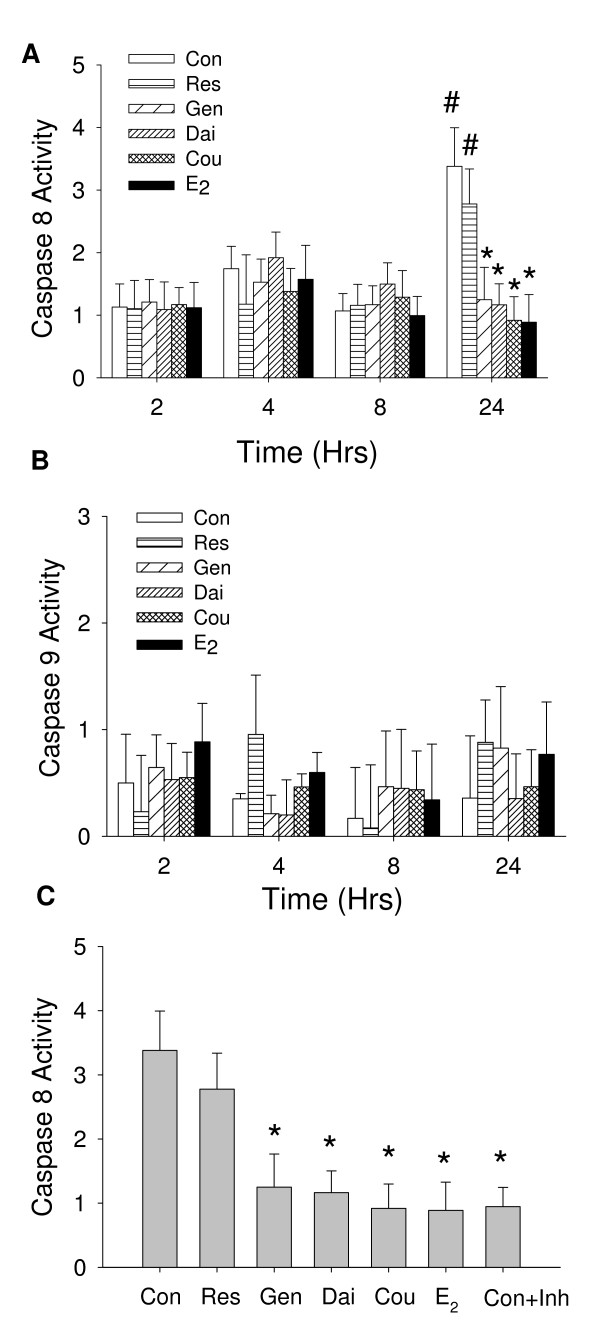
**The caspase activities of cells treated with phytoestrogens**. Caspase 8 and caspase 9 activities were measured in treated cells at the times indicated. Treatments were: vehicle control (Con), 10^-7 ^M *trans*-resveratrol (Res), 10^-7 ^M genistein (Gen), 10^-7 ^M Daidzein (Dai), 10^-8 ^M coumesterol (Cou), or 10^-9 ^M E_2_. We measured (A) caspase 8 or (B) caspase 9 (n = 8). * = p < 0.05 compared to control. # = p < 0.05 compared between 2 and 24 hr. (C) To demonstrate the specificity of these actions by phytoestrogens on caspase 8, caspase 8 activity was attenuated by 5 μM caspase 8 inhibitor Ac-IETD-CHO (Con+Inh) measured at 24 hours, (n = 16). * = p < 0.05 compared to control. The caspase activity is expressed as change in fluorescence intensity (excitation wavelength of 400 nm and an emission wavelength of 505 nm) during an hr for 2500 cells.

Cell numbers can also be increased by other mechanisms such as inhibition of apoptosis. Therefore, we measured the activities of both caspase-8 (of the extrinsic pathway) and caspase-9 (of the intrinsic pathway) in the cells treated with E_2 _or different phytoestrogens. After 24 hours of treatment, both the vehicle- and *trans*-resveratrol-treated cells increased caspase-8 activity. For E_2_-, coumesterol-, daidzein-, and genistein-treated cells, the caspase-8 activity remained unchanged (Figure 8A). Caspase-9 activity showed no significant changes with any treatment (Figure 8B). Inhibition of caspase-8 activity with specific antagonist, Ac-IETD-CHO in control cells confirmed the involvement of caspase-8 (Figure 8C).

### Activation of downstream targets of MAPKs by different phytoestrogens

**Figure 9:**
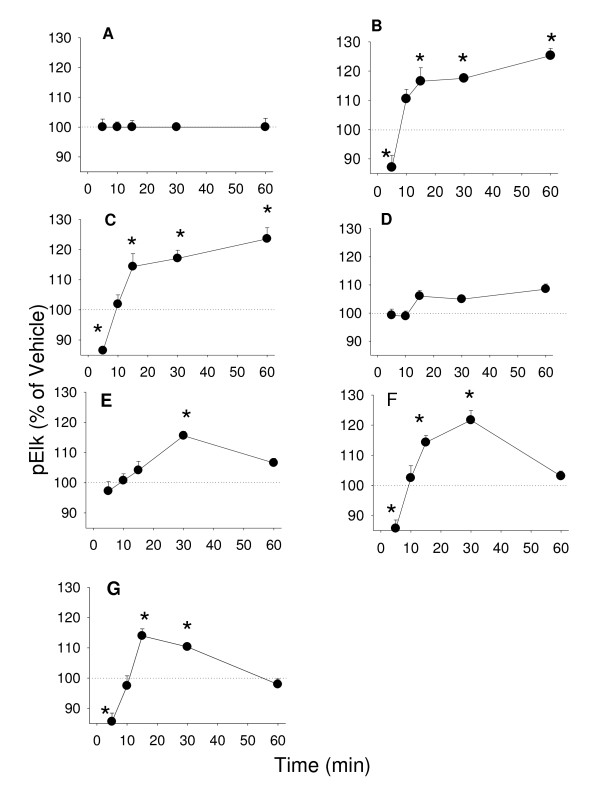
**The time course of estrogenic phospho-activation of pElk-1**. Time-dependent activation of Elk-1 by phosphorylation was measured by a quantitative plate immunoassay for phospho-Elk-1 (pELK-1) in cells treated with (A) 10^-7 ^M cholesterol (negative control), (B) 20 nM TPA (positive control), (C) 10^-9 ^M E_2_, (D) 10^-8 ^M coumesterol, (E) 10^-7 ^M daidzein, (F) 10^-7 ^M genistein, (G) 10^-7 ^M *trans*-resveratrol. * = p < 0.05 compared to vehicle only treated cells.

**Figure 10:**
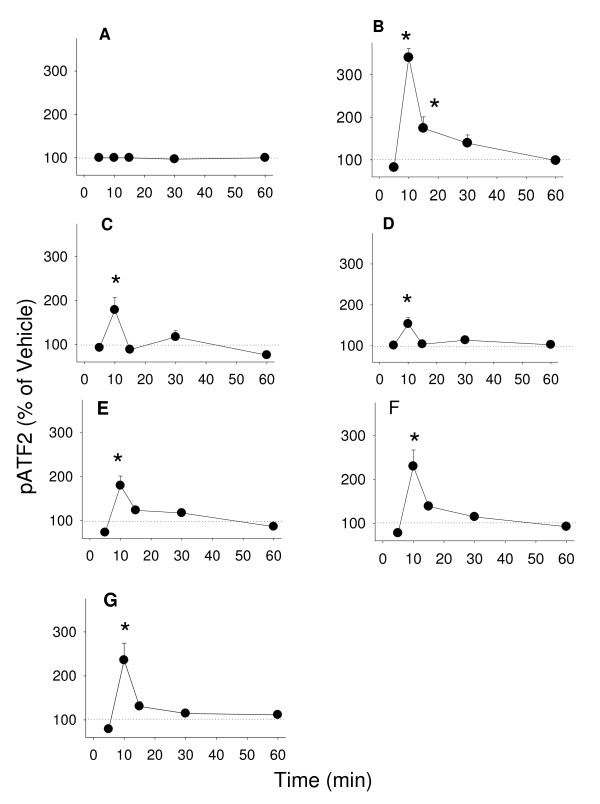
**The time course of estrogenic phospho-activation of pATF-2**. Time-dependent activation of ATF2 by phosphorylation was measured by a quantitative plate immunoassay for phospho-ATF-2 (pATF-2) in cells treated with (A) 10^-7 ^M cholesterol (negative control), (B) 20 nM TPA (positive control), (C) 10^-9 ^M E_2_, (D) 10^-8 ^M coumesterol, (E) 10^-7 ^M daidzein, (F) 10^-7 ^M genistein, (G) 10^-7 ^M *trans*-resveratrol. * = p < 0.05 compared to vehicle only treated cells.

Although the precise downstream events initiated by MAPKs which result in cell proliferation are not clearly understood, MAPKs are often shown to be part of the pathway leading to that function. We measured the phosphorylation levels of two well-known downstream targets for MAPKs: Elk-1 (Figure 9) and ATF-2 (Figure 10) resulting from treatment with E_2 _and different phytoestrogens. We observed time-dependent changes in Elk phosphorylation for most of these compounds at 15 min; daidzein was slower (30 min) and coumesterol failed to activate the transcription factor at all. Cells treated with 10^-9 ^M E_2 _showed a sustained elevated level of Elk-1 phosphorylation up to 60 minutes, as did the positive control activator TPA. Treatment with E_2_, TPA, and all phytoestrogens caused elevations of phosphorylated ATF-2 at 10 minutes followed by a rapid decline in all cases (Figure 10).

## Discussion

Cell proliferation is a complex process under tight regulation. Increasing cell number can be achieved by either speeding up progress through the cell cycle or by decreasing the amount or rate of cell death (frequently via the process of apoptosis for known hormonal effects). We demonstrated the proliferative effects of E_2_, and three of four different phytoestrogens (coumesterol, daidzein, genistein, but not *trans*-resveratrol) in a pituitary cell line which expresses high levels of membrane ERα, an experimental system well-suited to reveal the rapid actions of estrogens and compounds which mimic them, as we have done previously for other classes of estrogens [8,33-36]. Overall, our studies demonstrated that phytoestrogens can elicit nongenomic responses (rapid ERK, JNK, transcription factor and caspase activations) at concentrations expected to be achieved by dietary intake. The low concentrations at which these activations occur suggest that these nongenomic responses are relevant to common human exposures.

In our mERα-enriched pituitary tumor cell line, coumestrol, daidzein, and genistein increased cell proliferation with a lower potency than E_2_, but at concentrations spanning known serum or urine levels in humans [12-14,29]. However, *trans*-resveratrol did not change cell proliferation at any concentration tested, so it is unlikely to contribute to tumor cell growth at any exposure level, while the other phytoestrogens might do this, if present by themselves. However, lone exposure to phytoestrogens is unlikely because physiological estrogens are usually present across different life stages and for different sexes, spanning ~pM to nM concentrations [37]). Interestingly, 10^-7 ^M genistein treatment over 6-9 days was more robust in maintaining cell proliferation than a physiological concentration of E_2_. The response differences between closely related genistein and daidzein may be due to genistein's higher bioavailbility [12]. Specific blockage by the ERα/β antagonist ICI 182780 suggests that the proliferative effects of E_2_, coumesterol, daidzein, and genistein involve these ERs, most likely ERα for this cell line [8,38-40] since they are enriched for mERα and under our conditions do not appear to express ERβ.

We investigated co-treatments with E_2 _because these dietary estrogens have been suggested as foils for E_2_-mediated cell growth. Whether E_2 _was delivered at low or high physiological levels, all 4 tested phytoestrogens attenuated the proliferative effects of E_2_, including low doses of *trans*-resveratrol (≥ 10^-12 ^M) which did not cause proliferation by itself. The increased effectiveness at the lower phytoestrogen doses is reminiscent of the non-monotonic dose-responses that we have seen previously for nongenomic responses to a variety of estrogens [35,36]. Several possible mechanisms could be involved including competition for available ERs by the less potent phytoligands, differing changes in the ERs' affinity or activation [41], or activations of multiple but different signaling pathways which have different functional effects [13]. Whatever the mechanism, these data suggest that phytoestrogens at dietary levels may be able to combat the proliferative effects of the most prominent endogenous estrogen, E_2_, over a wide range of physiological concentrations.

One of the best-characterized rapid actions of estrogens is the phosphorylation of ERKs. The ERK pathway plays important roles in cellular processes like proliferation, differentiation, and survival. The oscillating and sustained stimulation patterns we showed for E_2 _and coumestrol, respectively, were similar to those previously observed by us and others [26,42]. Daidzein and genistein caused phosphorylation of ERK with similar early (5-10 min) peaks, while *trans*-resveratrol caused increased pERKs significantly only at a delayed (30 min) time point. These data suggest that phytoestrogens can cause differential (extent and pattern of oscillation) ERK activations, interwoven with probable deactivations by phosphatases [43]. The lack of early phase ERK activation by *trans*-resveratrol may be related to its lack of proliferative effects. This is in contrast to other reports that the later or sustained ERK peak is more important for correlations to cell proliferation [44].

All tested phytoestrogens elicited JNK activation, with only minor differences in timing, suggesting that the activation of this kinase was not responsible for differences in controlling cell numbers between these compounds. Phosphorylation of p38 could only be detected in E_2_-treated cells, so its actions also do not correlate with the proliferative abilities of these compounds. However, all three MAPKs that we studied are apparently involved in the signaling pathways important for GH_3_/B_6_/F_10 _cell proliferation, as inhibitors selective for each of these kinases attenuated cell proliferation, even in the vehicle control group (suggesting that other baseline or hormone-irrelevant growth controls involve these enzymes). For example, p38 has been related to bromocriptine-induced apoptosis in rat pituitary GH_3 _cells [45]. Since all MAPKs are involved, it is possible that they are differentially involved, based on the timing of the oscillating phases of kinase activation, to regulate cell proliferation [46]. However, all phytoestrogens inhibited proliferation when in combination with E_2_. Perhaps this is related to the ability of all of them to induce JNK phosphorylation with a similar time course pattern. In addition, these phytoestrogens could compete with E_2 _for binding to ERs and their roles in the same proliferation pathways.

The significant changes we see in MAPK activities are small, but many biological responses are probably modified in such small but significant increments, and they are probably no less important than big changes for dialing up and down functions. Our techniques for measuring these responses have now improved (quantitative plate immunoassays as opposed to Westerns), so that we can finally measure the significance of these small changes [10]. MAPKs receive lots of different signal inputs from multiple pathways in cells, and adjust to each with a new level of activity. Multiple small changes can add up to large ones, with MAPKs essentially acting as a summation device or a "rheostat". Activation of these kinases can cause either immediate functional responses, and/or give rise to eventual transcription of genes via phosphorylation of transcription factors, so some of these effects could be downstream genomic actions.

Phytoestrogen-induced MAPK activations could lead to downstream transcriptional events which might also influence proliferative responses, such as we observed for the phosphorylation of two known targets for MAPKs: Elk-1 and ATF-2 [22,23]. Our data showed that two types of time-dependent MAPK activations can be correlated with Elk-1 and ATF-2 phosphorylations. ERK activation just preceded or was coincident with the activations of Elk-1, as would be expected if the activated kinase then affects Elk-1 downstream. JNK activation was coincident with the beginning of Elk-1 phosphorylation and could participate in the subsequent (>15 min) Elk-1 phosphorylations. ATF-2 phosphorylation being very fast and brief in all cases is likely only affected by estrogen-induced ERK activity. However, the high levels of phosphorylated ATF-2 may suggest that other estrogen-induced mechanisms also contribute, such as the rise in calcium levels, also known to affect ATF-2 activation [47,48].

*Trans*-resveratrol, a phytoestrogen which did not cause cell proliferation on its own, increased phosphorylation of ERKs with a delayed response, while its JNK, p38, Elk-1, and ATF-2 responses did not differentiate it from the other estrogens. This suggests that ERK activation parameters were the most crucial for resveratrol's unique antiproliferative and perhaps pro-apoptotic (see below) actions. It will be interesting in the future to discover which other functional responses these activations may influence.

We further investigated the specific apoptotic pathways involved in regulating cell number by separately assessing the abilities of phytoestrogens to activate caspase-8 and caspase-9, pivotal enzymes in the intrinsic and extrinsic apoptosis pathways, respectively. Many types of cell treatments can induce apoptosis, including removal of sera from culture media, which can activate caspase-8, but does not involve caspase-9 [49]. Estradiol and phytoestrogens (except *trans*-resveratrol) suppressed the activation of caspase-8. The ability of resveratrol to inhibit the increase in cell numbers caused by E_2_, though it could not itself induce cell proliferation, could be due to its ability to allow this apoptotic pathway to remain activated. It is interesting that it is the extrinsic pathway which traditionally involves receptors for extracellular ligands presented at the cell membrane that is shown to be involved by our studies. In combination with the rapid activation of enzymes of the apoptotic pathway, this suggests a role for a membrane form of steroid receptor, as our previous studies have shown to be true for estrogenic responses in this cell line [50-53] and for glucocorticoid-induced apoptosis in lymphoid cell lines [54].

## Conclusion

Diet and nutrition are known to contribute to different rates of cancer progression [1,55]. Our study has provided evidence that different phytoestrogens can induce unique cell number regulation effects whose contributing signaling mechanisms can be multiple and complex. These different outcomes are probably cell type- and condition-dependent, so our data will add to the body of knowledge that must be accumulated in order to map out those differences. However, it is clear that some of these mechanisms are rapid and are likely to be mediated by membrane receptors for estrogens, which in the case of our model system is likely to be mERα. It is possible that phytoestrogens, with their similar yet unique signaling properties might be utilized as anti-tumor treatments, as we and others have shown that they can oppose both mechanisms and outcomes elicited by physiological estrogens. At the same time women may benefit from some of the other positive estrogenic signaling outcomes if phytoestrogens are used as postmenopausal replacement estrogens. *Trans*-resveratrol seems to be particularly promising in this regard based on our studies. Investigators are now beginning to decipher the component signaling pathways involved, so that we may eventually make these therapeutic choices based on scientific evidence.

## Competing interests

The authors declare that they have no competing interests.

## Authors' contributions

YJJ carried out the all the experiments in these studies. YJJ and CSW both participated in the design of the study and statistical analyses. All authors read and approved the final manuscript.

## Pre-publication history

The pre-publication history for this paper can be accessed here:

http://www.biomedcentral.com/1471-2407/9/334/prepub

## References

[B1] Cornwell T Cohick W Raskin I Dietary phytoestrogens and health Phytochemistry 2004 65 995 1016 10.1016/j.phytochem.2004.03.005 15110680

[B2] Brownson DM Azios NG Fuqua BK Dharmawardhane SF Mabry TJ Flavonoid effects relevant to cancer J Nutr 2002 132 3482S 3489S 12421874 10.1093/jn/132.11.3482S

[B3] Fritz WA Coward L Wang J Lamartiniere CA Dietary genistein: perinatal mammary cancer prevention, bioavailability and toxicity testing in the rat Carcinogenesis 1998 19 2151 2158 10.1093/carcin/19.12.2151 9886571

[B4] Gooren LJ Giltay EJ Bunck MC Long-term treatment of transsexuals with cross-sex hormones: extensive personal experience J Clin Endocrinol Metab 2008 93 19 25 10.1210/jc.2007-1809 17986639

[B5] Harris DM Besselink E Henning SM Go VLW Heber D Phytoestrogens Induce Differential Estrogen Receptor Alpha- or Beta-Mediated Responses in Transfected Breast Cancer Cells Experimental Biology and Medicine 2005 230 558 568 16118406 10.1177/153537020523000807

[B6] Whitten PL Lewis C Russell E Naftolin F Phytoestrogen influences on the development of behavior and gonadotropin function Proceedings of the Society for Experimental Biology & Medicine 1995 208 82 86 10.3181/00379727-208-43836 7892301

[B7] Maggiolini M Vivacqua A Fasanella G Recchia AG Sisci D Pezzi V The G protein-coupled receptor GPR30 mediates c-fos up-regulation by 17beta-estradiol and phytoestrogens in breast cancer cells J Biol Chem 2004 279 27008 27016 15090535 10.1074/jbc.M403588200

[B8] Bulayeva NN Wozniak A Lash LL Watson CS Mechanisms of membrane estrogen receptor-{alpha}-mediated rapid stimulation of Ca2+ levels and prolactin release in a pituitary cell line Am J Physiol Endocrinol Metab 2005 288 E388 E397 15494610 10.1152/ajpendo.00349.2004

[B9] Pappas TC Gametchu B Watson CS Membrane estrogen receptor-enriched GH_3_/B6 cells have an enhanced non-genomic response to estrogen Endocrine 1995 3 743 749 10.1007/BF03000207 21153164

[B10] Bulayeva NN Gametchu B Watson CS Quantitative measurement of estrogen-induced ERK 1 and 2 activation via multiple membrane-initiated signaling pathways Steroids 2004 69 181 192 15072920 10.1016/j.steroids.2003.12.003 PMC1201430

[B11] Bickoff EM Booth AN Lyman RL Livingston AL Thompson CR Deeds F Coumestrol, a new estrogen isolated from forage crops Science 1957 126 969 970 13486041 10.1126/science.126.3280.969-a

[B12] Mustafa AM Malintan NT Seelan S Zhan Z Mohamed Z Hassan J Phytoestrogens levels determination in the cord blood from Malaysia rural and urban populations Toxicology and Applied Pharmacology 2007 222 25 32 17490695 10.1016/j.taap.2007.03.014

[B13] Whitten PL Patisaul HB Cross-species and interassay comparisons of phytoestrogen action [Review] Environ Health Perspect 2001 109 5 20 11250801 10.1289/ehp.01109s15 PMC1240538

[B14] Adlercreutz H Fotsis T Lampe J Wahala K Makela T Brunow G Quantitative determination of lignans and isoflavonoids in plasma of omnivorous and vegetarian women by isotope dilution gas chromatography-mass spectrometry Scand J Clin Lab Invest Suppl 1993 215 5 18 8392221 10.3109/00365519309090693

[B15] Gehm BD McAndrews JM Chien PY Jameson JL Resveratrol, a polyphenolic compound found in grapes and wine, is an agonist for the estrogen receptor Proc Natl Acad Sci USA 1997 94 14138 14143 9391166 10.1073/pnas.94.25.14138 PMC28446

[B16] Walle T Hsieh F DeLegge MH Oatis JE Jr Walle UK HIGH ABSORPTION BUT VERY LOW BIOAVAILABILITY OF ORAL RESVERATROL IN HUMANS Drug Metab Dispos 2004 32 1377 1382 15333514 10.1124/dmd.104.000885

[B17] Hernandez M Soto-Cid A Rojas F Pascual L randa-Abreu G Toledo R Prostate response to prolactin in sexually active male rats Reproductive Biology and Endocrinology 2006 4 28 16707016 10.1186/1477-7827-4-28 PMC1524775

[B18] Torner L Toschi N Pohlinger A Landgraf R Neumann ID Anxiolytic and anti-stress effects of brain prolactin: Improved efficacy of antisense targeting of the prolactin receptor by molecular modeling Journal of Neuroscience 2001 21 3207 3214 11312305 10.1523/JNEUROSCI.21-09-03207.2001 PMC6762563

[B19] Wiklund J Wertz N Gorski J A comparison of estrogen effects on uterine and pituitary growth and prolactin synthesis in F344 and Holtzman rats Endocr 1981 109 1700 1707 10.1210/endo-109-5-1700 7297500

[B20] Gorski J Wendell D Gregg D Chun TY Estrogens and the genetic control of tumor growth. [Review] [[23] refs] Progress in Clinical & Biological Research 1997 396 233 243 9108601

[B21] Sarkar DK Hentges ST De A Reddy RH Hormonal control of pituitary prolactin-secreting tumors Front Biosci 1998 3 d934 d943 9696884 10.2741/a334

[B22] Hommes DW Peppelenbosch MP van Deventer SJH Mitogen activated protein (MAP) kinase signal transduction pathways and novel anti-inflammatory targets Gut 2003 52 144 151 12477778 10.1136/gut.52.1.144 PMC1773518

[B23] Santen RJ Song RX Masamura S Yue W Fan P Sogon T Adaptation to estradiol deprivation causes up-regulation of growth factor pathways and hypersensitivity to estradiol in breast cancer cells Adv Exp Med Biol 2008 630 19 34 18637482 10.1007/978-0-387-78818-0_2 PMC2641021

[B24] Marino M Galluzzo P Ascenzi P Estrogen signaling multiple pathways to impact gene transcription Curr Genomics 2006 7 497 508 18369406 10.2174/138920206779315737 PMC2269003

[B25] Santen RJ Song RX McPherson R Kumar R Adam L Jeng MH The role of mitogen-activated protein (MAP) kinase in breast cancer J Steroid Biochem Mol Biol 2002 80 239 256 11897507 10.1016/s0960-0760(01)00189-3

[B26] Bulayeva NN Watson CS Xenoestrogen-induced ERK 1 and 2 activation via multiple membrane-initiated signaling pathways Environ Health Perspect 2004 112 1481 1487 15531431 10.1289/ehp.7175 PMC1325963

[B27] Belcher SM Zsarnovszky A Estrogenic actions in the brain: estrogen, phytoestrogens, and rapid intracellular signaling mechanisms J Pharmacol Exp Ther 2001 299 408 414 11602649

[B28] Cato AC Nestl A Mink S Rapid actions of steroid receptors in cellular signaling pathways Sci STKE 2002 2002 RE9 12084906 10.1126/stke.2002.138.re9

[B29] Ho KJ Liao JK Nonnuclear actions of estrogen Arterioscler Thromb Vasc Biol 2002 22 1952 1961 12482819 10.1161/01.atv.0000041200.85946.4a

[B30] Watson CS Jeng YJ Kochukov MY Nongenomic actions of estradiol compared with estrone and estriol in pituitary tumor cell signaling and proliferation The FASEB Journal 2008 22 3328 3336 18541692 10.1096/fj.08-107672 PMC2518256

[B31] Zhou JH Yu DV Cheng J Shapiro DJ Delayed and persistent ERK1/2 activation is required for 4-hydroxytamoxifen-induced cell death Steroids 2007 72 765 777 17714751 10.1016/j.steroids.2007.06.007 PMC3655899

[B32] Gibson MK Nemmers LA Beckman WC Jr Davis VL Curtis SW Korach KS The mechanism of ICI 164,384 antiestrogenicity involves rapid loss of estrogen receptor in uterine tissue Endocr 1991 129 2000 2010 10.1210/endo-129-4-2000 1915080

[B33] Bulayeva N Gametchu B Watson CS Xenoestrogens can activate MAP kinases in a pituitary tumor cell line FASEB Summer Conference on Steroid Receptors in the Plasma Membrane [Snow Mass, CO] 2002 Ref Type: Abstract

[B34] Watson CS Bulayeva NN Wozniak AL Alyea RA Xenoestrogens are potent activators of nongenomic estrogenic responses PMCID:17174995 Steroids 2007 72 124 134 17174995 10.1016/j.steroids.2006.11.002 PMC1862644

[B35] Watson CS Alyea RA Jeng YJ Kochukov MY Nongenomic actions of low concentration estrogens and xenoestrogens on multiple tissues PMCID:17601655 Mol Cell Endocrinol 2007 274 1 7 17601655 10.1016/j.mce.2007.05.011 PMC1986712

[B36] Watson CS Jeng YJ Kochukov MY Nongenomic actions of estradiol compared with estrone and estriol in pituitary tumor cell signaling and proliferation FASEB J 2008 22 3328 3336 18541692 10.1096/fj.08-107672 PMC2518256

[B37] Greenspan FS Gardner DG Greenspan FS, Gardner DG Appendix: Normal Hormone Reference Ranges Basic and Clinical Endocrinology 2004 7 New York: Lange Medical Books, McGraw Hill 925 926

[B38] Watson CS Norfleet AM Pappas TC Gametchu B Rapid actions of estrogens in GH_3_/B6 pituitiary tumor cells via a plasma membrane version of estrogen receptor-∀ Steroids 1999 64 5 13 10323667 10.1016/s0039-128x(98)00107-x

[B39] Watson CS Zivadinovic D Bulayeva N Hawkins B Campbell CH Gametchu B Watson CS A membrane form of estrogen receptor-α mediates estrogenic, nongenomic effects The Identitities of Membrane Steroid Receptors 2003 Boston: Kluwer Academic Publishers 11 19

[B40] Watson CS Bulayeva NN Wozniak AL Finnerty CC Signaling from the membrane via membrane estrogen receptor-alpha: estrogens, xenoestrogens, and phytoestrogens PMCID:15862819 Steroids 2005 70 364 371 15862819 10.1016/j.steroids.2005.03.002

[B41] Kuiper GG Carlsson B Grandien K Enmark E Haggblad J Nilsson S Comparison of the ligand binding specificity and transcript tissue distribution of estrogen receptors alpha and beta Endocr 1997 138 863 870 10.1210/endo.138.3.4979 9048584

[B42] Alblas J Slager-Davidov R Steenbergh PH Sussenbach JS van der BB The role of MAP kinase in TPA-mediated cell cycle arrest of human breast cancer cells Oncogene 1998 16 131 139 9467952 10.1038/sj.onc.1201485

[B43] Zivadinovic D Watson CS Membrane estrogen receptor-alpha levels predict estrogen-induced ERK1/2 activation in MCF-7 cells Breast Cancer Res 2005 7 R130 R144 15642162 10.1186/bcr959 PMC1064105

[B44] Murphy LO Smith S Chen RH Fingar DC Blenis J Molecular interpretation of ERK signal duration by immediate early gene products Nat Cell Biol 2002 4 556 564 12134156 10.1038/ncb822

[B45] Kanasaki H Fukunaga K Takahashi K Miyazaki K Miyamoto E Involvement of p38 mitogen-activated protein kinase activation in bromocriptine-induced apoptosis in rat pituitary GH3 cells Biol Reprod 2000 62 1486 1494 10819748 10.1095/biolreprod62.6.1486

[B46] Chuang SM Wang IC Yang JL Roles of JNK, p38 and ERK mitogen-activated protein kinases in the growth inhibition and apoptosis induced by cadmium Carcinogenesis 2000 21 1423 1432 10874022

[B47] Ban N Yamada Y Someya Y Ihara Y Adachi T Kubota A Activating transcription factor-2 is a positive regulator in CaM kinase IV-induced human insulin gene expression Diabetes 2000 49 1142 1148 10909971 10.2337/diabetes.49.7.1142

[B48] Jeng YJ Kochukov MY Watson CS Membrane estrogen receptor-alpha-mediated nongenomic actions of phytoestrogens in GH3/B6/F10 pituitary tumor cells J Mol Signal 2009 4 2 19400946 10.1186/1750-2187-4-2 PMC2679742

[B49] Schamberger CJ Gerner C Cerni C Caspase-9 plays a marginal role in serum starvation-induced apoptosis Experimental Cell Research 2005 302 115 128 15541731 10.1016/j.yexcr.2004.08.026

[B50] Campbell CH Watson CS A comparison of membrane vs. intracellular estrogen receptor-∀ in GH3/B6 pituitary tumor cells using a quantitative plate immunoassay PMCID:11522334 Steroids 2001 66 727 736 11522334 10.1016/s0039-128x(01)00106-4

[B51] Norfleet AM Thomas ML Gametchu B Watson CS Estrogen receptor-α detected on the plasma membrane of aldehyde-fixed GH3/B6/F10 rat pituitary cells by enzyme-linked immunocytochemistry PMCID:10433242 Endocr 1999 140 3805 3814 10.1210/endo.140.8.6936 10433242

[B52] Norfleet AM Clarke C Gametchu B Watson CS Antibodies to the estrogen receptor-α modulate prolactin release from rat pituitary tumor cells through plasma membrane estrogen receptors PMCID:10627290 FASEB J 2000 14 157 165 10627290 10.1096/fasebj.14.1.157 PMC1189731

[B53] Wozniak AL Bulayeva NN Watson CS Xenoestrogens at picomolar to nanomolar concentrations trigger membrane estrogen receptor-alpha-mediated Ca2+ fluxes and prolactin release in GH3/B6 pituitary tumor cells PMCID:15811834 Environ Health Perspect 2005 113 431 439 15811834 10.1289/ehp.7505 PMC1278483

[B54] Gametchu B Watson CS Correlation of membrane glucocorticoid receptor levels with glucocorticoid-induced apoptotic competence using mutant leukemic and lymphoma cells lines Journal of Cellular Biochemistry 2002 87 133 146 12244567 10.1002/jcb.10288

[B55] Go VL Butrum RR Norman HA Review of the International Research Conference on Food, Nutrition, and Cancer, 2005 J Nutr 2005 135 2925S 2926S 16317151 10.1093/jn/135.12.2925S

